# Photoperiod does not affect thermal acclimation of shoot-scale gas exchange but is important for shoot development in cuttings of Norway spruce (*Picea abies* (L.) H. Karst)

**DOI:** 10.1093/treephys/tpaf112

**Published:** 2025-09-16

**Authors:** Astrid Fridell, Göran Wallin, Curt Almqvist, Lasse Tarvainen

**Affiliations:** Department of Biological and Environmental Sciences, University of Gothenburg, PO Box 463, Gothenburg SE-405 30, Sweden; Department of Biological and Environmental Sciences, University of Gothenburg, PO Box 463, Gothenburg SE-405 30, Sweden; College of Agriculture, Forestry and Food Sciences, University of Rwanda, PO Box 210, Musanze, Rwanda; The Forestry Research Institute of Sweden (Skogforsk), Uppsala Science Park, Uppsala SE-751 83, Sweden; Department of Biological and Environmental Sciences, University of Gothenburg, PO Box 463, Gothenburg SE-405 30, Sweden

**Keywords:** boreal forests, bud development, climate change, day length, photosynthesis, warming

## Abstract

The growth of boreal trees is expected to benefit from increasing global temperatures through enhanced photosynthetic rates and longer growing seasons. However, since photoperiod is independent of climate change, it may limit the expected growth benefits from a longer growing season and could thus constrain boreal trees’ physiological responses to warming. We carried out a growth chamber experiment on 2-year-old Norway spruce (*Picea abies*) cuttings from two latitudinal origins to investigate the interaction between day length (20/4 h vs 14/10 h light/dark) and enhanced temperatures (25/20 °C vs 15/10 °C day/night) on height growth, bud development and shoot-scale gas exchange. Height growth was greater under longer day length while bud development occurred faster both under longer day length and higher growth temperature. Growth temperature did not have a significant effect on the light-saturated photosynthetic rate but higher growth temperature resulted in lower dark respiration rate. Cuttings in the low-growth temperature treatment exhibited higher apparent quantum yields indicating that lower growth temperature benefited net carbon uptake under low light availability, such as the conditions experienced by seedlings growing in the forest understory. Day length did not influence the thermal acclimation of shoot-scale gas exchange. The two populations from different origins did not differ in the measured parameters, except for a higher dark respiration rate in the high latitude cuttings. Overall, while day length did not affect the thermal acclimation of photosynthetic processes, it appears to constrain height growth and bud development, thereby reducing the potential performance benefit of a warming-induced lengthening of the growing season.

## Introduction

Forest net carbon uptake is increasing due to rising temperatures and atmospheric CO_2_ concentrations (e.g., Bastos et al. 2019, Ciais et al. 2019), with the temperature increase being especially high in the arctic and boreal regions (IPCC 2021). The responses of trees to further warming will depend on multiple factors including the ability for physiological thermal acclimation (Way and Oren 2010, Stinziano and Way 2014, Reich et al. 2022, Dusenge et al. 2023) and sensitivity to photoperiod (Bauerle et al. 2012, Stinziano and Way 2014, Mu et al. 2023).

Boreal coniferous forests are commonly expected to benefit from the warmer climate because higher spring and winter temperatures can speed up the recovery from winter dormancy, reduce the risk for cold-induced damage and allow for earlier bud burst and later growth cessation, thereby increasing the length of the growing season (Burke et al. 1976, Kramer et al. 2000, Hyvönen et al. 2007, Slaney et al. 2007, Wallin et al. 2013). In addition, boreal trees currently operate below their optimum temperature and their photosynthetic carbon uptake is expected to respond positively to warming (e.g., Hall et al. 2009, 2013, Wallin et al. 2013, Dusenge et al. 2023). However, continued warming may result in a decline in photosynthesis and growth if boreal trees are unable to acclimate to higher temperatures (Tranquillini and Havranek 1985, Stinziano and Way 2014, D’Orangeville et al. 2018, Reich et al. 2022). Thermal acclimation of photosynthetic biochemistry is mainly achieved by shifting the photosynthetic optimum temperature and thereby adjusting the maximum rates of carboxylation (*V*_cmax_) and electron transport (*J*_max_) to maximize gross carbon uptake at the new growth temperature (Berry and Björkman 1980, Yamori et al. 2010). While the ability for photosynthetic acclimation varies among species and growth environments (Björkman et al. 1975, Yamori et al. 2010), it is well-documented that plants are not able to fully thermally acclimate their photosynthesis to a change in growth temperatures (e.g., Yamori et al. 2014, Sendall et al. 2015, Kumarathunge et al. 2019, Crous et al. 2022, Dusenge et al. 2023). Notably, a meta-analysis by Way and Yamori (2014) showed that evergreen woody species, like boreal conifers, were especially limited in their ability for photosynthetic thermal acclimation to improve their performance at higher temperatures. In addition to changes in photosynthetic capacity, plant respiration is expected to increase exponentially with elevated temperature. Differences in the thermal responses between photosynthesis and respiration could affect the balance between carbon uptake and release and lead to reduced net photosynthetic rates (Berry and Björkman 1980, Slot and Kitajima 2015). However, respiratory downregulation is a common acclimation response to elevated growth temperatures (e.g., Tjoelker et al. 1999, Dusenge et al. 2019, Ren et al. 2024), which may at least partly compensate for photosynthetic limitations and allow for maintaining similar net photosynthetic rates over a wide range of growth temperatures (Dusenge et al. 2019). Although respiratory thermal acclimation has been observed in boreal conifers such as Scots Pine (*Pinus sylvestris*), the capacity for it is weak in spruce (*Picea*) species (Stinziano et al. 2015, Kurepin et al. 2018, Dusenge et al. 2021).

As a practical way for dealing with potential reductions in tree performance caused by climate change, it has been suggested that introduction of trees of a southern origin could enhance the acclimation capacity of quantitative traits, such as photosynthetic capacity and growth, to a warming climate (e.g., Oleksyn et al. 2001, Partanen et al. 2004, Aitken and Whitlock 2013, Hamilton et al. 2016). Supporting this, it has been shown that bud development is faster, and in some cases starts earlier or continues longer, in individuals from warmer origins in provenance studies on several conifer species (Oleksyn et al. 2001, Hamilton et al. 2016, Milesi et al. 2019, Svystun et al. 2021). In addition, Milesi et al. (2019) reported that Norway spruce individuals from a southern origin grew better than those of local (Swedish) origin in a study comparing the growth performance of trees from seven genetic clusters. It should be noted, however, that these benefits are likely to occur only as long as the increasing growth temperatures remain below the photosynthetic thermal optima of the introduced southern provenances.

Like warming, photoperiod can affect phenology and photosynthetic capacity and may therefore constrain responses to warming (Busch et al. 2007, Bauerle et al. 2012, Stinziano and Way 2014). This constraint is especially important to account for in studies using latitude gradients as warming analogues to investigate the physiological and growth responses to elevated temperatures (e.g., Huang et al. 2010, De Frenne et al. 2013, Diao et al. 2023). Overall, phenology responds to both temperature and photoperiodic signals (Cooke et al. 2012) with the importance of each cue varying among species. The differences in photoperiodic sensitivity may depend on the species’ adaptive strategy, prioritizing either protection against unfavorable seasons or the efficient use of growing resources (Leinonen and Hänninen 2002). Photoperiod-sensitive species enter and exit winter dormancy partly in response to day length, which may afford protection against cold-induced damage. However, this strategy does not allow them to take full advantage of the prolongation of the growing season in response to warming (Battey 2000, Stinziano and Way 2014, Marchin et al. 2015, Way and Montgomery 2015). Furthermore, photoperiodic sensitivity varies depending on latitude origin (Hamilton et al. 2016) and photoperiod-sensitive trees growing at higher latitudes generally respond to photoperiodic changes in the autumn earlier than trees of the same species from lower latitudes (Vaartaja 1954, Partanen 2004). In boreal species, photoperiod can both affect the timing of bud burst (Partanen et al. 2001, Basler and Körner 2012) and modify the temperature response of growth cessation (Heide 1974, Stinziano et al. 2015, Hamilton et al. 2016). In general, the growing season has been extended more during spring than during the autumn, suggesting that photoperiod plays a larger role in entering winter dormancy than in recovering from it (Barichivich et al. 2013, Way and Montgomery 2015, Richardson et al. 2018). Although the seasonal nature of light availability in the temperate and boreal regions strongly limits photosynthesis, especially during the autumn when temperatures are still relatively high (e.g., Bauerle et al. 2012, Tarvainen et al. 2015, Stangl et al. 2022), the interactive response of photosynthetic capacity to photoperiod and warming has been studied less than the phenological response. Most studies on boreal species have focused on the autumn decline and have reported that the photosynthetic warming response is generally not constrained by photoperiod (Bourdeau 1959, Stinziano et al. 2015, Hamilton et al. 2016, Stinziano and Way 2017) but Busch et al. (2007) observed that light-saturated net CO_2_ assimilation was sensitive to photoperiod in Jack pine (*Pinus banksiana* Lamb.) seedlings exposed to increased autumn temperatures. Given the limited number of studies and their contrasting results, it is still unclear to what extent photoperiod directly affects the physiological thermal acclimation capacity of boreal trees and, thereby, their ability to perform well in a warmer climate.

This study aimed to determine the effect of photoperiod on the temperature response of height growth, bud development and photosynthesis in Norway spruce to improve predictions of boreal forests’ responses to future climate. The following research questions were investigated:

(i) Does photoperiod modify the temperature response of height growth and bud development?
(ii) Does photoperiod affect the thermal acclimation of photosynthetic capacity and/or net photosynthesis?
(iii) Does tree origin affect the growth and physiological responses to photoperiod and temperature?

## Materials and methods

### Plant material and experimental setup

This study was performed on two-year-old cuttings of Norway spruce (*Picea abies* (L.) H. Karst) from two origins, central European and Swedish/Russian-Baltic, included in the Norway spruce breeding program in southern Sweden (Chen et al. 2019, Milesi et al. 2019). The cuttings were collected in the field from the forestry research institute of Sweden’s (Skogforsk) research station in Ekebo (55°95’N, 13°11’E) in early February 2021. The cuttings were initially frozen and were allowed to thaw and release from winter dormancy in growth chambers with a start-up program (Start-up, Table 1) before being exposed to the treatments. The cuttings were planted in a peat-perlite mix (S-jord, Hasselfors garden, Örebro, Sweden) in 1.5-L pots and kept well-watered. In addition, they received nutrients throughout the experiment including nitrogen, phosphorus, potassium (with an NPK ratio of 5:1:4), and the most essential minerals (boron, copper, iron, manganese, molybdenum, zinc and magnesium). Weeds and insects were manually removed from the pots and plants when discovered.

**Table 1:** Summary of the temperature and day length treatments used in the study.

**Treatment**	**Temperature (°C)**		**Day length (h)**		***T***_**d**_ **(°C)**	***Q***_**d**_ **(mol m**^**−2**^ **day**^**−1**^**)**
	Day	Night	Day	Night		
Start-up	10	5	12	12	7.5	7.5
LT-SD	15	10	14	10	12.9	8.7
LT-LD	15	10	20	4	14.2	12.5
HT-SD	25	20	14	10	22.9	8.7
HT-LD	25	20	20	4	24.2	12.5

Abbreviations: HT = High growth temperature, LT = low growth temperature, LD = long day, and SD = short day.

After 2 weeks in Start-up, 24 plants from each origin were randomly divided into four treatments consisting of two growth temperatures (*T*_growth_) and two day lengths. Daytime temperatures were set to 15 and 25 °C, and nighttime temperatures were set to 10 and 20 °C under the low-temperature (LT) and the high-temperature (HT) conditions, respectively. Day length was either 14 h of light (06:00–20:00 h) and 10 h of darkness under the short-day (SD) conditions, or 20 h of light (03:00–23:00 h) and 4 h of darkness under the long-day (LD) conditions. This resulted in four combinations of treatments, LT-SD, LT-LD, HT-SD and HT-LD, where the daily sum of photosynthetic photon flux density (*Q*_d_) differed between the two day length treatments and the daily mean temperature (*T*_d_) differed between all treatments as a result of a combination of different temperatures and day lengths (Table 1). This in turn, resulted in different temperature sums (*T*_sum_ = *T*_d_ × Days of treatment) in all treatments over the 3-month study period. Day lengths of 14–20 h, defined as the time between sunrise and sunset, occur at 62°N in the mid-boreal forest zone between approximately April 5 and September 5.

To avoid chamber effects, the cuttings and the treatment they belonged to were rotated between the four growth chambers once a week throughout the experiment. The growth chambers had a light intensity, expressed as photosynthetic photon flux density, of 173 ± 48 μmol m^−2^ s^−1^ and a relative humidity of 74.8 ± 6.2%. The light intensity experienced by the cuttings in the growth chambers was low compared with saturating light levels but is similar to the mostly diffuse radiation reaching the understory of mature Norway spruce stands (e.g., Tarvainen et al. 2015).

### Height growth and bud development

Height growth and bud development were measured on all 48 plants included in the experiment at the end of the 3-month treatment period. Height growth is an important selection trait in the Swedish Norway spruce breeding program (Sonesson and Eriksson 2003) and was therefore used as an indicator of growth responses in this study. The growth of the cuttings was determined by measuring their total aboveground height before and after the treatment period.

Bud development was monitored on terminal and whorl buds and was categorized as an average of the whole plant. Monitoring was conducted approximately every third day during the experiment. The visible development of the buds was categorized based on the changes in bud scales (Slaney et al. 2007), according to a six-stage classification system devised by (Langlet 1960) and modified by Krutzsch (1973) and Hannerz (1999). The development stages span from A (buds enclosed by needles and not visible unless the needles are parted) to E (a longer brush of needles has emerged, and all bud scales have disappeared) as described in Slaney et al. (2007). Buds in all treatments had the average development stage of A before the start of the experiment. For this study, the time to complete bud burst was recorded at stage E, which was used to calculate the *T*_sum_ from the beginning of treatment.

### Gas exchange measurements

After 3 months of exposure to treatment conditions, photosynthetic response to light and intercellular CO_2_ concentration (*A*–*C*_i_ curves) were measured on a single one-year-old fully developed shoot (i.e., the bud burst occurred the year before the experiment) from eight randomly selected plants from each treatment, with four plants randomly chosen from the high latitude group and four from the low latitude group. The measurement was conducted on 7.5 cm long shoot segments using two LI6400 portable photosynthesis systems with conifer chambers (Li-Cor inc., Lincoln, NE, USA). The shoots were prepared by removing needles at the points where the shoot axis passes the chamber gasket. Leak tests were carried out for each measured shoot and sticky tack was used to seal eventual leaks. The gas exchange measurements were conducted between 10:00 and 17:00 h on the measurement days. All measurements were made at 25 °C and the relative humidity was kept between 60 and 80% to avoid stomatal closure (Lange et al. 1971).

Light response curves were measured first, following a dark acclimation period of 30 min. The CO_2_ level was set to 415 p.p.m., near the current ambient level (IPCC 2021). Net photosynthesis was measured using a light intensity series of 0, 20, 40, 60, 80, 200, 400, 700, 1000 and 1500 μmol m^−2^ s^−1^ photosynthetically active radiation (PAR). The light response curves were used to determine light-saturated photosynthesis (*A*_sat_), apparent quantum yield (AQY) and dark respiration (*R*_d_). The *A*–*C*_i_ curves were measured to determine the photosynthetic capacity parameters, apparent *V*_cmax_ and apparent *J*_max_, on the same shoots in saturating light (1000 μmol m^−2^ s^−1^), determined from the light response curves. Photosynthesis at different CO_2_ levels was measured using a concentration series of 415, 60, 110, 170, 250, 400, 600,1200, 1500 and 2000 μmol mol^−1^. The measurements were done using the *A*–*C*_i_ curve autoprogram with a minimum wait time of 120 s and a maximum wait time of 240 s, within this time period a measurement was made when the following stability criteria were met: rate of change (linear slope) in the sample cell [CO_2_] < 1 μmol mol^−1^, [H_2_O] < 0.5 mmol mol^−1^ and flow rate <1 μmol s^−1^ averaged over 15 s. Empty chamber measurements were used to correct for the effect of diffusion into and out of the measurement chamber at low respectively high CO_2_ concentrations.

### Needle properties

The measured 1-year-old shoots were harvested after completed measurements and placed into a −20 °C freezer until determination of needle area and dry mass. The projected needle area was measured using scanned images (Epson 1600+ equipped for dual scanning) of the needles and WinSEEDLE Pro 5.1a (Regent Instruments, Canada) analysis software. Needle dry mass was determined after drying at 70 °C for 48 h, and leaf mass per area (LMA) was calculated from the dry mass and the projected needle area. Mass-based needle nitrogen content (*N*_m_) was determined by a SerCon 20-22 infrared ratio mass spectrometer coupled with an elemental analyzer (Sercon Ltd, Crewe, UK). Area-based needle nitrogen content (*N*_area_) was calculated from LMA and *N*_m_.

### Data analysis and statistics

Curve-fitting was done using R version 4.1.1 (R Core Team 2021). The light response curves were fitted using the model from Marshall and Biscoe (1980) with the R package ‘photosynthesis’ using the ‘fit_aq_response’ function (Stinziano et al. 2020). To determine the apparent *V*_cmax_ and *J*_max_ from the *A*–*C*_i_ curves the model described by Farquhar et al. (1980) was used. This was done in the R package ‘plantecophys’ using the ‘fitacis’ function with the ‘bilinear’ fitting model (Duursma 2015). Estimates of the Michaelis–Menten constants for CO_2_ and O_2_ (K_c_ and K_o_) as well as the CO_2_ compensation point (Γ^*^) were provided with a built-in function of leaf temperature following Bernacchi et al. (2001). Furthermore, the temperature response functions of Bernacchi et al. (2001, 2003) were used to scale the apparent *V*_cmax_ and *J*_max_ to 25 °C (*V*_cmax25_, *J*_max25_) to facilitate comparison with previously reported values.

All statistical tests were done using IBM SPSS Statistics 29 (IBM Corporation, Armonk, NY, USA). The Shapiro–Wilk test was used to test for normal distribution, and Levene’s test was used to test for homogeneity of variance of the data. Data that did not meet the normality and/or the homogeneity criteria were transformed to meet the assumptions of the statistical tests. Output data from the fitted light response and *A*–*C*_i_ curves, height growth, bud development, LMA and needle N content were initially analyzed as dependent variables in a three-way analysis of variance (ANOVA) with the origin, *T*_growth_ and day length as fixed independent variables. However, because tree origin was not a significant factor for the studied responses, data from the two provenance groups were pooled and analyzed further using two-way ANOVAs with *T*_growth_ and day length as fixed independent variables (except for *R*_d_).

## Results

### Height growth and bud development

**Figure 1:**
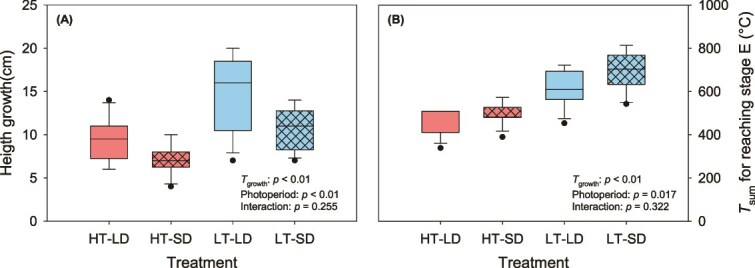
(A) Height growth (cm) during the experiment. (B) The temperature sum (T_sum,_ °C) needed to reach the last shoot development stage. Line inside the box is the median, lower and upper box borders show the 25th and 75th percentiles, the whiskers indicate the 10th and 90th percentiles and the dots indicated potential outliers (*n* = 12). For description of the treatments, see Table 1.

Low *T*_growth_ resulted in greater height growth under both photoperiod treatments (*P* < 0.01; Figure 1A). In addition, LDs resulted in greater height growth under both *T*_growth_ (*P* < 0.01; Figure 1A). Both *T*_growth_ and photoperiod significantly affected bud development (Figure 1B). The *T*_sum_ required for complete bud burst was larger under low *T*_growth_ compared with high *T*_growth_ under both photoperiods (*P* < 0.01; Figure 1B). Furthermore, plants grown under SD conditions required a larger *T*_sum_ to reach complete bud burst compared with plants grown under LD conditions (*P* = 0.017; Figure 1B). The largest among-treatment differences in bud development occurred during the early stages, A and B1 (see Figure S1 available as Supplementary Data at *Tree Physiology* Online). No significant interactions between *T*_growth_ and photoperiod were detected for the total height growth or the bud development (*P* ≥ 0.26; Figure 1A and B).

### Gas exchange and photosynthetic capacity

**Figure 2:**
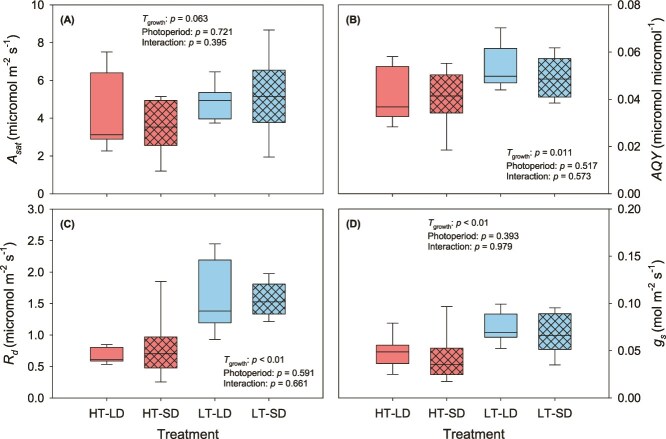
Effect of growth temperature and day length on (A) light-saturated photosynthesis (A_sat_, μmol m^−2^ s^−1^), (B) apparent quantum yield (AQY, μmol CO_2_ μmol^−1^ PPFD), (C) dark respiration (R_d_, μmol m^−2^ s^−1^) and (D) stomatal conductance (g_s_, mol m^−2^ s^−1^) at 25 °C. Line inside the box is the median, lower and upper box borders show the 25th and 75th percentiles, and the whiskers indicate the highest and lowest values (*n* = 8). For description of the treatments, see Table 1.

Light-saturated net photosynthesis did not respond to *T*_growth_ or photoperiod (*P* = 0.06 and *P* = 0.72, respectively; Figure 2A). AQY was higher in the cuttings from the low *T*_growth_ treatment (*P* = 0.011; Figure 2B) but did not respond to photoperiod (*P* = 0.52; Figure 2B). Dark respiration was significantly higher in plants grown in low *T*_growth_ for both photoperiod treatments (*P* < 0.01; Figure 2C), whereas photoperiod did not significantly affect *R*_d_ (*P* = 0.59; Figure 2C). In addition, *R*_d_ was the only variable for which plant origin had a significant main effect in the three-way ANOVA with the plants from higher latitude having higher *R*_d_ compared with the low-latitude plants (*P* = 0.024). Stomatal conductance was significantly higher in low *T*_growth_ plants (*P* < 0.01; Figure 2D) while photoperiod did not significantly affect *g*_s_ (*P* = 0.39). No significant photoperiod × *T*_growth_ interactions were found for *A*_sat_, AQY, *R*_d_ or *g*_s_ (*P* > 0.39; Figure 2A–D).

**Figure 3:**
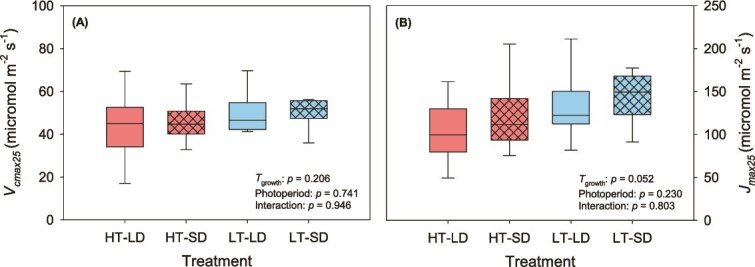
Effect of growth temperature and day length on (A) the apparent maximum rate of carboxylation at 25 °C (*V*_cmax25_, μmol m^−2^ s^−1^) and (B) the apparent maximum rate of electron transport at 25 °C (*J*_max25_, μmol m^−2^ s^−1^). Line inside the box is the median, lower and upper box borders show the 25th and 75th percentiles, and the whiskers indicate the highest and lowest values (*n* = 8). For description of the treatments, see Table 1.

Neither *T*_growth_ nor photoperiod significantly affected *V*_cmax25_ or *J*_max25_ (*P* ≥ 0.21 and *P* ≥ 0.052, respectively; Figure 3A and B). In addition, no significant *T*_growth_ × photoperiod interactions were observed for the photosynthetic capacity responses (*P* > 0.80; Figure 3A and B).

### Needle properties

**Figure 4:**
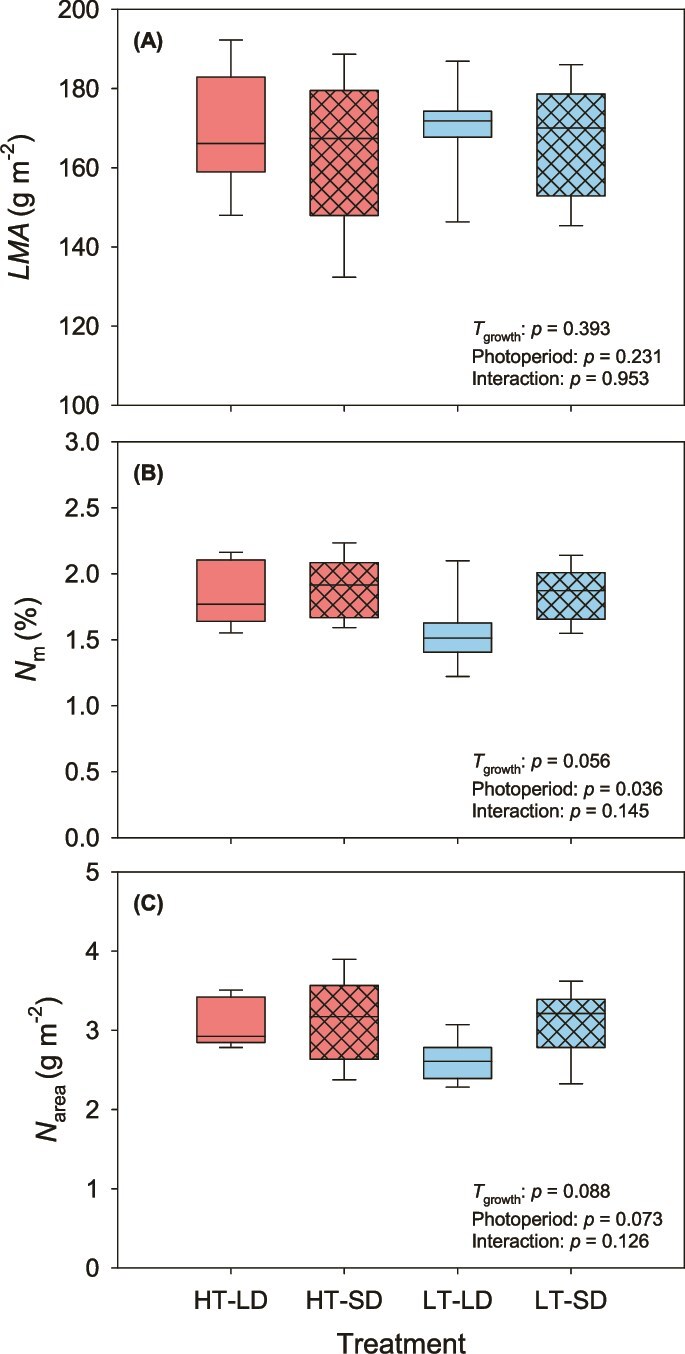
Effect of growth temperature and day length on (A) leaf mass per area (*LMA*, g m^−2^), (B) mass-based nitrogen content (*N*_m_, %) and (C) area-based nitrogen content (*N*_area_, g m^−2^). Line inside the box is the median, lower and upper box borders show the 25th and 75th percentiles, and the whiskers indicate the highest and lowest values (*n* = 8). For description of the treatments, see Table 1.

Neither *T*_growth_ nor photoperiod significantly affected LMA (*P* = 0.39 and *P* = 0.23, respectively; Figure 4A). Mass-based needle nitrogen content was higher in cuttings in the short-day treatments (*P* = 0.036; Figure 4B), while no effect of *T*_growth_ on *N*_m_ was found (*P* = 0.056). When expressed on an area-basis needle nitrogen content did not vary significantly with either *T*_growth_ or photoperiod or (*P* = 0.09 and *P* = 0.07, respectively; Figure 4C). No significant *T*_growth_ × photoperiod interactions were observed for any of the measured needle properties (*P* > 0.12; Figure 4A–C).

## Discussion

### Effect of growth temperature and photoperiod on height growth and bud development

The studied Norway spruce cuttings grew taller in lower *T*_growth_, which matches the results from a meta-analysis on 58 tree species, with the majority of the included studies using potted trees, that found that growth was reduced in treatments with a temperature above 13 °C (Way and Oren 2010) and with other studies showing that increases in *T*_growth_ reduce growth in boreal forest trees (e.g., Kivimäenpää et al. 2013, Chen and Luo 2015, D’Orangeville et al. 2018, Reich et al. 2022). In this study, height growth was overall reduced under the short photoperiod with this effect being stronger in low *T*_growth_, which suggests that at high *T*_growth_ the temperature constraint dominated the growth response. Similarly, short photoperiod has been found to lead to earlier growth cessation and lower total growth in other studies on boreal conifers (e.g., Partanen et al. 2004, Stinziano et al. 2015, Hamilton et al. 2016, Stinziano and Way 2017).

We found bud development to be faster under high *T*_growth_, which agrees with results from earlier studies (e.g., Hänninen et al. 2007, Schleip et al. 2008, Hall et al. 2009, Olsen et al. 2014). Slaney et al. (2007) observed that bud burst in mature Norway spruce trees occurred 2–3 weeks earlier in whole-tree chambers at a spring-time elevated *T*_growth_ of 3–4 °C but also that a higher *T*_sum_ was required for bud burst in elevated temperature than in ambient temperature. This contrasts the results from this study where we found that the *T*_sum_ required for completing the bud development was higher for the low *T*_growth_ cuttings. This difference may be partly due tree age, which is known to affect the growth rhythm including the shoot elongation period in Norway spruce (Ununger et al. 1988), and also due to the use of constant *T*_growth_ and photoperiod in this study, compared with the overall lower *T*_growth_ and gradual increases in both temperature and photoperiod that followed the natural seasonal cycle, including warmer and cooler periods, used by Slaney et al. (2007). Furthermore, the results from this study show that bud development is slower under shorter photoperiod, which is consistent with previous findings regarding the spring phenology of boreal and alpine species including Norway spruce (e.g., Partanen et al. 1998, 2004, Basler and Körner 2012). The slower bud development would lead to the new shoots in the short photoperiod treatments reaching their carbon break-even point later (Hall et al. 2009), thereby reducing their contribution to plant-scale net carbon uptake, which may in part explain why the cuttings exposed to shorter photoperiod grew less during the experiment.

### Effect of growth temperature and photoperiod on gas exchange

Studies on thermal acclimation of net photosynthesis, commonly measured in saturating light (*A*_sat_), have found evidence of both increasing and decreasing rates, partly depending on species and how the chosen treatment temperatures relate to a given species’ photosynthetic thermal optimum (Way and Yamori 2014). In Norway spruce, previous studies have similarly yielded variable results regarding the thermal acclimation of *A*_sat_, including reduced rates at high *T*_growth_ (Kurepin et al. 2018), no acclimation (Stinziano et al. 2015, Lamba et al. 2018) and reduced rates at low *T*_growth_ during spring recovery (Tranquillini and Havranek 1985, Wallin et al. 2013). In this study, we observed no thermal acclimation response for *A*_sat_ but AQY was higher in the low *T*_growth_ cuttings. Because *R*_d_ was reduced in the high *T*_growth_ cuttings, a common acclimation response (e.g., Atkin and Tjoelker 2003, Atkin et al. 2005, Slot and Kitajima 2015, Lamba et al. 2018), and the *V*_cmax25_ and *J*_max25_ were similar for cuttings from both *T*_growth_ treatments, the difference in AQY is likely due to the downregulation of stomatal conductance under high *T*_growth_. This would reduce the CO_2_ available for carboxylation reactions in the high *T*_growth_ cuttings, causing net photosynthesis to decrease, as was also suggested regarding *A*_sat_ reduction at high *T*_growth_ in black spruce (Dusenge et al. 2020) and mature Norway spruce (Lamba et al. 2018). Given the low light conditions in the growth chambers in this study, the observed increase in AQY by lower *T*_growth_ could in part explain why the low *T*_growth_ cuttings were able to grow taller than the cuttings in the high *T*_growth_ treatment. Notably, the AQY reduction in high *T*_growth_ contrasts the results of Wallin et al. (2013) who observed higher AQY under elevated temperatures in field-grown mature Norway spruce during spring recovery. However, the study by Wallin et al. (2013) used lower *T*_growth_ (ambient and slightly elevated northern Swedish spring temperatures) compared with this study (15 and 25 °C) where high *T*_growth_ treatment may have been close to or above the temperature optimum for net photosynthesis. Furthermore, the importance of temperature (relative to other environmental factors) in determining net photosynthesis in field-grown Norway spruce has been shown to be the greatest during the spring months (Bergh et al. 1998, Tarvainen et al. 2015), which may further explain the difference between the observations made during spring recovery by Wallin et al. (2013) and this study where the AQY was determined after 3 months of exposure to higher *T*_growth_.

Photosynthetic capacity, *V*_cmax_ and *J*_max_, is well-known to increase with growth temperature until the temperature optimum is reached (e.g., Way and Yamori 2014, Kumarathunge et al. 2019). A study on mature, field-grown Norway spruce found the optimum temperature of *V*_cmax_ to be 35.3 °C, while *J*_max_ peaked at 26.5 °C (Tarvainen et al. 2013), close to the daytime temperature in the high *T*_growth_ treatments. The observed capacity values at a reference temperature, *V*_cmax25_ and *J*_max25_ (Figure 3), were similar to those reported for Norway spruce previously (Tarvainen et al. 2013, Stinziano et al. 2015, Lamba et al. 2018). Previous studies on acclimation of *V*_cmax25_ (Stinziano et al. 2015, Lamba et al. 2018, Yang et al. 2020) and *J*_max25_ (Stinziano et al. 2015) have reported these parameters to be insensitive to *T*_growth_ in Norway spruce, which agrees with the findings of this study. However, Stinziano et al. (2015) observed a decline in *V*_cmax25_ and *J*_max25_ in growth temperatures below 8 °C, indicating that elevated temperature could delay the autumn decline of photosynthesis. Dusenge et al. (2020) reported a decline in *V*_cmax_ and *J*_max_ at a reference temperature with warming in black spruce (*Picea mariana*) and suggested that it was related to a decline in needle nitrogen content. However, no significant change in needle nitrogen content with *T*_growth_ was observed in this study.

While photosynthetic capacity has been found to respond to photoperiod in some cases (Busch et al. 2007, Bauerle et al. 2012), the overall photoperiodic sensitivity varies among species (Marchin et al. 2015). In this study, we found that photoperiod did not affect the thermal acclimation of *A*_sat_, AQY or *R*_d_ in either *T*_growth_, which agrees with previous results on boreal species including Norway spruce during the autumn decline period (Bourdeau 1959, Stinziano et al. 2015, Hamilton et al. 2016, Stinziano and Way 2017). As with all other studied variables, we also found no significant interaction between photoperiod and *T*_growth_ with respect to photosynthetic capacity. It should be noted that a longer photoperiod could result in an acclimation response for two reasons, the duration of illumination itself (day length) and the increase in the accumulated PAR (= day length × light intensity). While we are unaware of any studies that have separated these two factors when investigating the interactive effects of warming and photoperiod on physiological acclimation in trees, studies on crops have suggested that the combination of a longer photoperiod and lower light intensity improves carbon uptake under warming compared with a shorter photoperiod and higher light intensity (Elkins and van Iersel 2020, Jeong et al. 2025). Given the low light intensity, representative of forest understory conditions, in the growth chambers used in this study, it is unlikely that the observed responses were affected by the photosynthetic system being overwhelmed by saturating light intensities. In nature, light availability is a key driver of the seasonality of net photosynthesis in boreal trees (e.g., Tarvainen et al. 2015, Stangl et al. 2022), and it has been shown that light limitation results in a decline in net photosynthesis in Norway spruce during the autumn independent of *T*_growth_ (Stinziano et al. 2015).

### Effect of tree origin

The two populations included in this study exhibited similar rates of height growth and bud development, which differs from previous observations on conifers of faster bud development in individuals belonging to southern provenances (Oleksyn et al. 2001, Hamilton et al. 2016, Milesi et al. 2019, Svystun et al. 2021). However, it should be noted that as this short study only included individuals from two provenances it is not possible to use its results to generalize regarding provenance effects. In fact, Milesi et al. (2019) investigated growth and bud development in 6 to 15-year-old field-grown Norway spruce trees from seven different origins, including the two provenances used in this study, grown in southern Sweden and found that trees with a southern origin grew taller and wider than trees of local origin. Similar results were presented by Partanen (2004) who studied the growth performance of Norway spruce provenances along a latitude gradient in Finland and found southern provenances to grow more, possibly because the northern trees entered winter dormancy earlier as a mechanism to avoid cold-induced damage.

The effect of provenance on conifer species’ physiological acclimation responses to warming has not been studied extensively. Hamilton et al. (2016) reported that while both photoperiod and *T*_growth_ significantly affect the photosynthetic capacity of white spruce (*Picea glauca*) seedlings, provenance does not, which is consistent with the findings of this study. However, we observed reduced *R*_d_ in the lower latitude cuttings under low *T*_growth_, which is likely due to a long-term adaptive response to growth in warmer conditions in their original habitat.

## Conclusions

This study focused on the potential interactions between photoperiod and growth temperature on the thermal acclimation of height growth, bud development and photosynthesis of Norway spruce cuttings. Height growth benefited from low growth temperature and long photoperiod, whereas bud development occurred faster under high growth temperature and long photoperiod. Light-saturated net photosynthesis and photosynthetic biochemistry exhibited limited potential to acclimate to both growth temperature and photoperiod, while AQY was downregulated under high growth temperature. We found no effect of provenance on the acclimation of growth or photosynthesis but note that a provenance effect on bud development and growth is commonly observed in long-term field studies. Overall, the results of this study suggest that while it is important to account for photoperiod when modelling bud development and growth of Norway spruce forests, photoperiod does not appear to be an important factor for photosynthetic thermal acclimation.

## Supplementary Material

Supplementary_data_Fridell_et_al_second_revision_tpaf112

## Data Availability

The data underlying this article are available under a reserved DOI in the researchdata.se digital repository at doi:10.5878/pjgb-yy92.
