# Supplementary material for: Photoperiod does not affect thermal acclimation of shoot-scale gas exchange but is important for shoot development in cuttings of Norway spruce (Picea abies (L.) H. Karst)
Source: Tree Physiol. 2025 Sep 16;45(12):tpaf112. doi: 10.1093/treephys/tpaf112 (PMC12704415; doi:10.1093/treephys/tpaf112)
Supplement: Supplementary_data_Fridell_et_al_second_revision_tpaf112 [file supplementary_data_fridell_et_al_second_revision_tpaf112.docx]

**Photoperiod does not affect thermal acclimation of shoot-scale gas exchange but is important for shoot development in cuttings of Norway spruce (*Picea abies* (L.) H. Karst)**

Astrid Fridell^a^, Göran Wallin^a^, Curt Almqvist^b^, Lasse Tarvainen^a,*^

^a^Department of Biological and Environmental Sciences, University of Gothenburg, PO Box 461, Gothenburg SE-405 30, Sweden

^b^The Forestry Research Institute of Sweden (Skogforsk), SE-751 83, Uppsala, Sweden

***Supplementary information***

To examine the treatment effects on bud development, the bud development stages were given numerical values as follows: A = 0, B1 = 5, B2 = 10, C = 20, D = 30 and E = 40 following the method of Hannerz (1999). Furthermore, because buds developed at different rates among the individuals in a given treatment, the average bud development stage against the temperature sum (*T*_sum_ ) within a treatment was evaluated using the following scale, presented by Slaney et al (2007): A < 2.5; B1 ≥ 2.5, < 7.5; B2 ≥ 7.5, <15; C ≥ 15, <25; D ≥ 25, <35; E ≥ 35. The largest among-treatment differences in bud development occurred during the early stages, A and B1 (Supplementary Figure S1, below).

**References**

Hannerz M. 1999. Evaluation of temperature models for predicting bud burst in Norway spruce. Can J For Res, 29(1):9-19. https://doi.org/10.1139/x98-175.

Slaney M, Wallin G, Medhurst J, Linder S. 2007. Impact of elevated carbon dioxide concentration and temperature on bud burst and shoot growth of boreal Norway spruce. Tree Physiol. 27(2):301-312. https://doi.org/10.1093/treephys/27.2.301.

**Figure S1** Mean stage of bud development with the temperature sum (T_sum_) for 2-year-old Norway spruce cuttings under long or short daylengths, and high or low growth temperatures. HT = High growth temperature, LT = low growth temperature, LD = long day, and SD = short day. Note that some stages were not observed in the average development data (B1 and D, under short days and high growth temperature, and B2 under long days and high growth temperature), although they were observed for the individual plants.
